# Stress-Related Herpesvirus Reactivation in Badgers Can Result in Clostridium Proliferation

**DOI:** 10.1007/s10393-021-01568-2

**Published:** 2021-12-06

**Authors:** Ming-shan Tsai, Chris Newman, David W. Macdonald, Christina D. Buesching

**Affiliations:** 1 Wildlife Conservation Research Unit, Department of Zoology, University of Oxford, Recanati-Kaplan Centre, Abingdon Road, Tubney House, Tubney, Oxfordshire, OX13 5QL UK; 2 Cook’s Lake Farming Forestry and Wildlife Inc (Ecological Consultancy), Queens County, NS Canada; 3 Department of Biology, Irving K. Barber Faculty of Science, The University of British Columbia, Okanagan, Kelowna, BC Canada

**Keywords:** gammaherpesvirus, sexually transmitted infection, wildlife, carnivora, one health, food-borne disease

## Abstract

*Clostridium perfringens* is an important food-borne zoonotic pathogen and a member of the commensal gut microbiome of many mammals. Predisposing factors such as coinfection with other pathogens or diet change can, however, cause overgrowth and subsequent disease development. Here we investigated the occurrence of *C. perfringens* in a free-ranging badger population with up to 100% prevalence of herpesvirus infection. Herpesvirus reactivation is known to be associated with increased susceptibility bacterial infections. PCR screening of rectal swabs from 69 free-ranging badgers revealed 15.9% (11/69, 95% CI = 9.1–26.3%) prevalence of detectable *C. perfringens* (Type A) DNA in the digestive tracts of assymptomatic animals. The results of Fisher’s exact test revealed *C. perfringens* detection was not biased by age, sex and seasons. However, badgers with genital tract gammaherpesvirus (MusGHV-1) reactivation (*p* = 0.007) and infection with a specific MusGHV-1 genotype (*p* = 0.019) were more prone to of *C. perfringens* proliferation, indicating coinfection biased dynamics of intestinal *C. perfringens*. An inclusion pattern analysis further indicated that, causally, MusGHV-1 reactivation potentiated *C. perfringens* detection. Whether or not specific MusGHV-1 genotype infection or reactivation plays a role in *C. perfringens* overgrowth or disease development in badgers will require further investigation. Nevertheless, a postmortem examination of a single badger that died of fatal disease, likely associated with *C. perfringens*, revealed MusGHV-1 detection in the small intestine.

**Supplementary Information:**

The online version contains supplementary material available at 10.1007/s10393-021-01568-2.

## Introduction

Most vertebrates test positive for one or several herpesvirus species (Shrawat et al. 2018) because the host immune system is unable to eradicate the virus from the body after primary infection. Instead, herpesviruses undergo a period of latency inside host cells but can reactivate when the host immune system is weakened (e.g., due to stress: (Seeber et al. 2018; Baumworcel et al. 2019) or coinfection with other pathogens: (Dai et al. 2020a)): For example, the two human gammaherpesvirus species, the Epstein–Barr virus (EBV, causing mononucleosis) and the Kaposi’s sarcoma-associated herpesvirus (KSHV), establish latency in lymphocytes after primary infection (Liang et al. 2009; Barton et al. 2011; Young et al. 2016; Johnson and Tarakanova 2020) and shed infectious virions into the oral cavity or genital tract when reactivated. The Equine gammaherpesviruses 2 and 5 (EHV-2 and EHV-5) are associated with abortion in mares, although the causal relationship remains unclear (Galosi et al. 2005; Marenzoni et al. 2013). In wild carnivores, gammaherpesviruses also produce pervasive, lifelong infection (e.g., felids: (Lozano et al. 2015; Tateno et al. 2017), musteloids: (King et al. 2004; Nicolas de Francisco et al. 2020), and ursids: (Black et al. 2019)). Repetitive reactivation, or active virus replication and shedding, happens asymptomatically but can occasionally cause recrudescent symptoms. These include pruritic (mucocutaneous) lesions or ulcers in the skin or genitalia, as well as neoplasms (Abade dos Santos et al., 2020; Gagnon et al., 2011; Nicolas de Francisco et al., 2020; Tsai, et al., 2020; Tseng et al., 2013). Although infections generally remain sub-patent, clinically severe disease can occur when host immunity is compromised due to senescence, stress responses, coinfections or neoplastic disease conditions (Sehrawat et al. 2018). One such comorbidity factor is the proliferation of *Clostridium perfringens*, as reported in Asian elephant calves (*Elephas maximus*) diagnosed with fatal *Elephant endotheliotropic herpesvirus* (EEHV) infection (Boonsri et al. 2018), and dairy cows (Frazier et al. 2002) and other captive artiodactyls (Flach et al. 2002) with *Bovine herpesvirus 4* (BHV-4) infection.

*C. perfringens* is a spore-forming gram-positive bacterium and a common member of mammals, birds and reptiles' commensal gut microbiome (Lyhs et al. 2013; Meng et al. 2017; Milton et al. 2017; Ramos et al. 2019; Razmyar et al. 2014). However, in some cases, it can result in pathogenic zoonotic infections (Weese and Staempfli 2000; Van Immerseel et al. 2004; Silva and Lobato 2015). Eating foods, particularly undercooked meat contaminated with *C. perfringens*, is a common source of food poisoning due to the bacterium’s ability to tolerate extreme high and low temperatures (Li and McClane 2006) and aerobic conditions (Briolat and Reysset 2002). If dysbiosis occurs and *C. perfringens* proliferates in the small intestine, watery diarrhea and gastroenteritis can develop into necrotic enteritis. It can also cause emphysematous cholecystitis in the gallbladder and fulminate gas gangrene (also known as clostridial myonecrosis, caused by *α*-toxins) in humans and other animals (Miyahara et al. 2013; Kiu and Hall 2018). Symptoms of *C. perfringens* food poisoning may include nausea, vomiting, abdominal pain and fever. Symptoms usually develop within 8–12 h but can take up to 24 h from ingestion. In extremis, *C. perfringens* can prove fatal in domestic animals and wildlife (Silva and Lobato 2015), although in human food poisoning cases it usually self-resolves within 24 h (Kiu and Hall 2018). The major virulence factor of *C. perfringens* arises from the secretion of various enterotoxins, which are used to classify the strain as Type A, B, C, D, E, F and G (Kiu and Hall 2018). The key enterotoxin of type A strain, also called the α-toxin, causes hemolysis of erythrocytes (Sakurai et al. 2004), cell death, necrosis (Navarro et al. 2018) and disintegration of tight junctions between epithelial cells in the gut (Morris et al. 2012). Infections will respond to various antibiotic treatments, where clindamycin, metronidazole, rifampin and tetracycline are more efficacious than penicillin.

*C. perfringens* has frequently been isolated from the feces of healthy wild animal species in captivity or the field. Severe patho-morbidity is rare, although mortality due to necrotic enteritis has been reported in captive wild mammals and birds, and—more rarely—also in the field (Asaoka et al. 2004; Butler et al. 2008; Silva and Lobato 2015; Gartrell et al. 2017). A 3-year study by Vierheilig et al. (2013) revealed higher prevalence and abundance of *C. perfringens* in the feces of Carnivora than of ruminant wildlife species; another study by Cox et al. (2005) reported similar findings. These results suggest diet plays an important role in the epidemiology of *C. perfringens* (Silva et al. 2014), and second, that carnivores (such as feral dogs, cats, fish-eating avian species, foxes or badgers), omnivores (wild boar or domestic chickens) or scavengers (vultures) (Meng et al. 2017) may be significant sources of environmental *C. perfringens* contamination. It is thus essential to monitor and identify risk factors associated with high prevalence/prolific shedding of *C. perfringens* in animal feces at the human–livestock–wildlife interface, in order to establish and track transmission routes, as well as contamination levels in food or water sources (Van Immerseel et al. 2004; Kiu and Hall 2018).

The *Mustelid gammaherpesvirus 1* (MusGHV-1) was first reported in 2002 (Banks et al. 2002) and confirmed to have an almost 100% prevalence in wild badger populations (King et al. 2004; Sin et al. 2014). MusGHV-1 infection is typically asymptomatic but results in a high occurrence of viral shedding in the genital tract (Kent et al. 2017). Genital MusGHV-1 reactivation in adults is linked to stressors (Tsai et al. 2021) and associated with impaired female reproductive capacity (Tsai et al. 2020). Although gammaherpesvirus reactivation generally involves no or only mild disease, it has been identified in other species as a predisposing factor for secondary bacterial infection or cancer development (Nordengrahn et al. 1996).

As part of an ongoing investigation into the causes and consequences of MusGHV-1 reactivation in the European badger, *Meles meles* (hereafter ‘badger’) (Tsai et al. 2021), here we examined demographic traits, seasons and coinfection with different strains of MusGHV-1 to identify risk factors associated with intestinal *C. perfringens* proliferation. We also report the results of a badger necropsy, where *C. perfringens* was abundant in the ileum, in Supplementary file 1.

## Materials and Methods

To assess the background prevalence of *C. perfringens* in the study population, we tested 69 rectal swabs collected from 50 badgers sampled in 3 seasons (spring, summer and autumn) in 2018 (for detailed trapping and sampling methods, please see Tsai et al., 2021). None of these sampled animals exhibited any clinical symptoms indicative of *C. perfringens* infection at the time of sampling. DNA from the swabs was extracted and purified based on the method described in Tsai et al (2020) and the Qiagen DNeasy Blood and Tissue Kit according to the manufacturer’s instruction. Purified DNA samples were screened using *C. perfringens* toxin genes-specific multiplex PCR (Baums et al. 2004) (Table 1). We also used a MusGHV-1 specific PCR (King et al., 2004; Tsai et al., 2020) targeting the DNA polymerase gene to detect MusGHV-1 DNA from the same rectal and genital swab samples. Based on substitutions in the partial MusGHV-1 DNA polymerase gene, two different MusGHV-1 genotypes circulate in our study population (Tsai et al. 2021). We determined the MusGHV-1 genotype for each individual with Sanger sequencing results of successfully amplified PCR products from genital swab samples (Tsai et al. 2021).

**Table 1:** Primer list used in this study.

Gene	Name	Primer (5′–3′)	Concentration (μM)	Length	References
*Clostridium perfringens multiplex PCR*					
cpa	CPA5L	AGTCTACGCTTGGGATGGAA	0.2	900	Baums et al. (2004)
	CPA5R	TTTCCTGGGTTGTCCATTTC	0.2		
cpb	CPBL	TCCTTTCTTGAGGGAGGATAAA	0.138	611	
	CPBR	TGAACCTCCTATTTTGTATCCCA	0.138		
cpe	CPEL	GGGGAACCCTCAGTAGTTTCA	0.067	506	
	CPER	ACCAGCTGGATTTGAGTTTAATG	0.067		
etx	CPETXL	TGGGAACTTCGATACAAGCA	0.046	396	
	CPETXR	TTAACTCATCTCCCATAACTGCAC	0.046		
iap	CPIL	AAACGCATTAAAGCTCACACC	0.083	293	
	CPIR	CTGCATAACCTGGAATGGCT	0.083		
cpb2	CPB2L	CAAGCAATTGGGGGAGTTTA	0.117	200	
	CPB2R	GCAGAATCAGGATTTTGACCA	0.117		
*Clostridium perfringens other toxin gene*					
NetB	NetB-F	CTTCTAGTGATACCGCTTCAC	0.6	738	Rood et al. (2018)
	NetB-R	CGTTATATTCACTTGTTGACGAAAG	0.6		
NetE	NetE-F	TAGAAAACGTTCAATTGTATGG	0.2	601	
	NetE-R	AGAAAGCGCTGATACAGCTAATAAA	0.2		
NetF	NetF-F	AACAATATGTACAGGTATAACT	0.2	862	Gohari et al. (2015)
	NetF-R	TTGATAGGTATAATATGGTTCT	0.2		
NetG	NetG-F	TTGTTCAGGATTAGTAGCATTA	0.2	860	
	NetG-R	CATGAGTTGCATAAGTTGGTGT	0.2		
*Clostridium difficile multiplex PCR*					
tcdA	tcdA-F	GCATGATAAGGCAACTTCAGTGGTAa	0.6	629	Persson et al. (2008)
	tcdA-R	AGTTCCTCCTGCTCCATCAAATG	0.6		
tcdB	tcdB-F	CCAAARTGGAGTGTTACAAACAGGTG	0.4	410	
	tcdB-R1	GCATTTCTCCATTCTCAGCAAAGTA	0.2		
	tcdB-R2	GCATTTCTCCGTTTTCAGCAAAGTA	0.2		
cdtA	cdtA-F1	GGGAAGCACTATATTAAAGCAGAAGC	0.05	221	
	cdtA-F2	CTGGGTTAGGATTATTTACTGGACCA	0.05		
	cdtA-R	GGGAAACATTATATTAAAGCAGAAGC	0.1		
ctdB	ctdB-F	TTGACCCAAAGTTGATGTCTGATTG	0.1	262	
	ctdB-R	CGGATCTCTTGCTTCAGTCTTTATAG	0.1		
16S rDNA	PS13	GGAGGCAGCAGTGGGGAATA	0.05	1062	
	PS14	TGACGGGCGGTGTGTACAAG	0.05		

**Figure 1:**
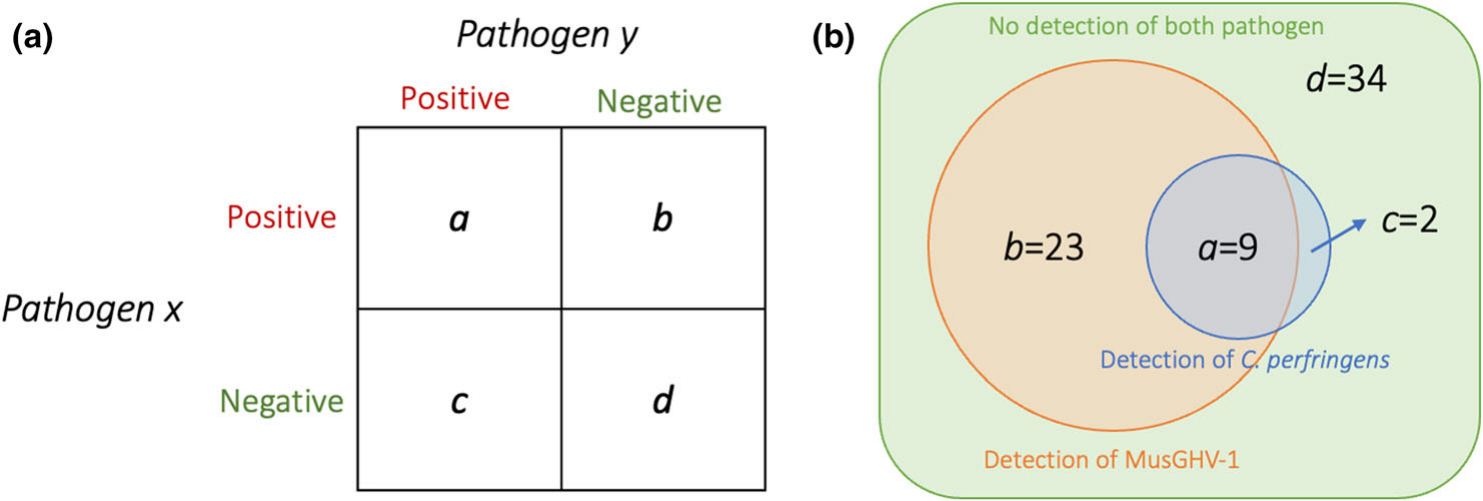
**a** A 2 × 2 table used for testing unequalness of observations (*a* = counts for individuals with positive detection of both pathogen *x* and *y*; *b* = counts for individuals with positive detection of pathogen *x* but negative for pathogen *y*; *c* = counts for individuals with negative detection of pathogen *x* but positive detection of pathogen *y*; *d* = counts for individuals with negative detection of both pathogen *x* and *y*). (Cavali-Sforza and Bodmer 1972). **b** Illustration of detection events of genital MusGHV-1 and *C. perfringens* of the sampled individuals (*n* = 68), where *C. perfringens* detection in gut is likely to be a consequence event of MusGHV-1 detection in genital tract.

We used Fisher’s exact test to identify the association between *C. perfringens* present in rectal swabs with sampling seasons, demographic traits, including sex and age group (juvenile: < 2 years old; young adults: 2 ≤ *x* < 5 years old; old adults: 5 ≤ *x* < 8 years old; very old adults: ≥ 8 years old), MusGHV-1 present in rectal/genital swabs and MusGHV-1 genotypes. We further applied an inclusion pattern analysis to explore the causal relationship between detection of MusGHV-1 and *C. perfringens*. We used the output derived from the equation $\frac{{\left( {b - c} \right)^{2} }}{{\left( {b + c} \right)}}$, which applied values taken from the diagonal of the *b* and *c* quadrants of the 2 × 2 table in Figure 1a. This output was then compared with the critical $\chi^{2}$ value at 1° of freedom with *p*-values of 0.05 and 0.001. An output higher than these critical $\chi^{2}$ values indicated a significant imbalanced diagonal quadrant. This would evidence that detection of either pathogen was not equally dependent and that one caused a predisposition to infection with the other; that is, when *b* is greater than *c* (or when *c* is greater than *b*), then infection of pathogen *x* is highly likely to predispose infection with pathogen *y* (or y is highly likely to predispose for *x*) (Cavali-Sforza and Bodmer 1972).

## Results

From the *C. perfringens* toxin genes-specific multiplex PCR results, in apparently asymptomatic animals the prevalence of *C. perfringens* was 15.9% (11/69, 95% CI = 9.1–26.3%), with all positive instances classified as type A.

We found no overall association between rectal swab MusGHV-1 and *C. perfringens* detection rate (Fisher’s exact test: *p* = 1), nor any effect of season, sex, or age (Table 2). However, we identified a strong positive correlation between genital MusGHV-1 detection and intestinal *C. perfringens* detection (Pearson’s r value = 0.31, Fisher’s exact test *p*-value = 0.019). We also identified an imbalance of observations in the opposite diagonal of the inclusion correlation matrix (Fig. 1 provides a 2 × 2 matrix of MusGHV-1 DNA detected in genital swabs (*x*) and *C. perfringens* DNA detected in rectal swabs (*y*), collected concurrently from the same individual, where *a* = 9, *b* = 23, *c* = 2, *d* = 34, Fig. 1b). This inclusion pattern analysis gave a *χ*^2^ of 17.64, which was much larger than the critical *χ*^2^ value at *p* > 0.05 (3.84, with1 degree of freedom) or at *p* > 0.001 (10.83, with1 degree of freedom). This indicated that *C. perfringens* detection was a consequence of MusGHV-1 reactivation. Thus, when *C. perfringens* was detected in the gut, MusGHV-1 was almost always reactivated; but when MusGHV-1 was reactivated, *C. perfringens* was not always detectable. Consequently, MusGHV-1 reactivation appeared to act as a predisposing factor for *C. perfringens* proliferation in badger intestines.

**Table 2:** Univariate analysis of *C. perfringens* risk factors.

Variable	C. p. Positive/total	Prevalence (%)	95% CI (%)	*p*-value
*Season*				
Spring	3/22	13.6	4.7–33.3	0.097
Summer	7/25	28.0	14.2–47.6	
Autumn	1/22	4.5	0.8–21.8	
*Sex*				
Male	4/33	12.1	4.8–27.3	0.337
Female	7/36	19.4	9.8–35	
*Age group*				
Juvenile	5/23	21.7	9.7–41.9	0.518
Young adult	3/16	18.8	6.6–43	
Old adult	0/8	0.0	0–32.4	
Very old adult	3/22	13.6	4.7–33.3	
*MusGHV-1 DNA in rectal swab*				
Positive	7/36	19.4	9.7–35	0.518
Negative	4/33	12.1	4.8–27.3	
*MusGHV-1 DNA in genital swab*				
Positive	9/32	28.1	15.6–45.4	0.019
Negative	2/36	5.6	1.5–18.1	
*MusGHV-1 genotype*				
Common	4/44	9.1	3.6–21.2	0.007
Novel	7/17	41.2	21.6–64	

Upon testing for any correlation between *C. perfringens* detection with either genotype, we found that *C. perfringens* occurrence was significantly more likely with the MusGHV-1 novel genotype, with a prevalence of 41.2% (7/17), compared to only 9.1% (4/44) in badgers infected with the MusGHV-1 common genotype (Fisher’s exact test: *p* = 0.007).

## Discussion

Our study demonstrates that *C. perfringens* detection was highly correlated with MusGHV-1 reactivation in the badgers we sampled. Furthermore, co-occurrence with *C. perfringens* was more likely to occur among individuals infected with the MusGHV-1 novel genotype. This shows that different individuals within the same population can have markedly different pathogen profiles and risks of disease pathogenesis. Although we did not investigate the directionality of causation empirically (whether the bacterium caused viral reactivation or viral reactivation compromised immunity, allowing bacterial proliferation to detectable levels), the inclusion pattern we found (Fig. 1b) suggested that it is MusGHV-1 reactivation that promotes *C. perfringens* overgrowth.

**Figure 2:**
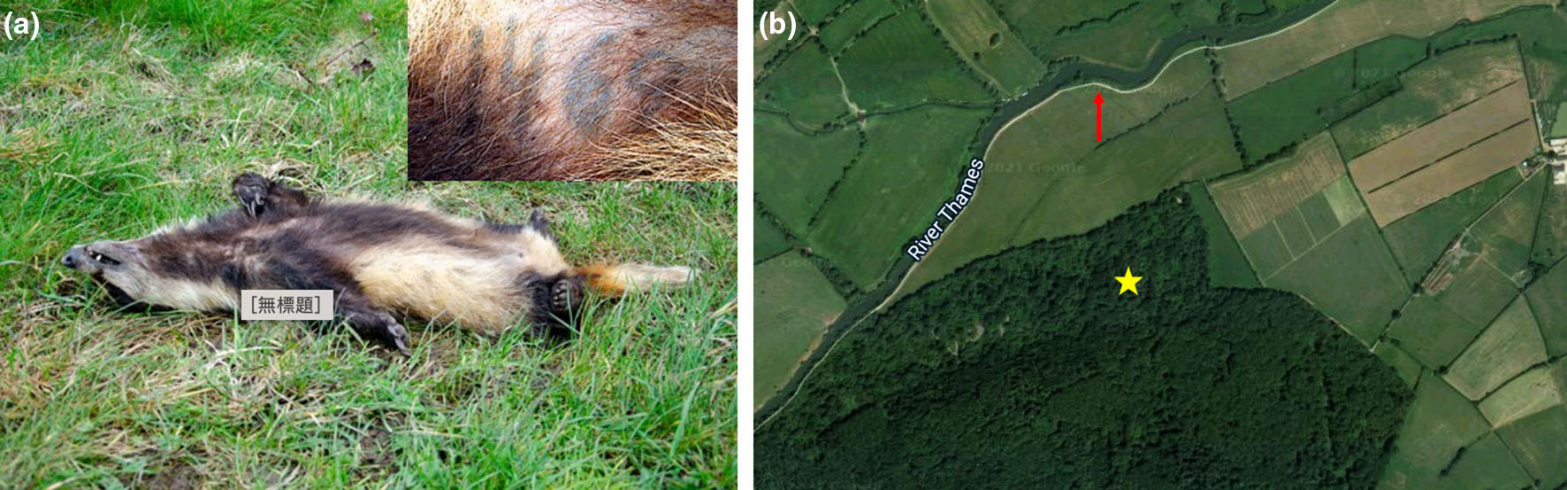
The female badger with tattoo number 1469 (**a**) found dead on a grassland near the River Thames (red arrow) about 770 m from her set of residency (yellow star) (**b**) (Color figure online).

**Figure 3:**
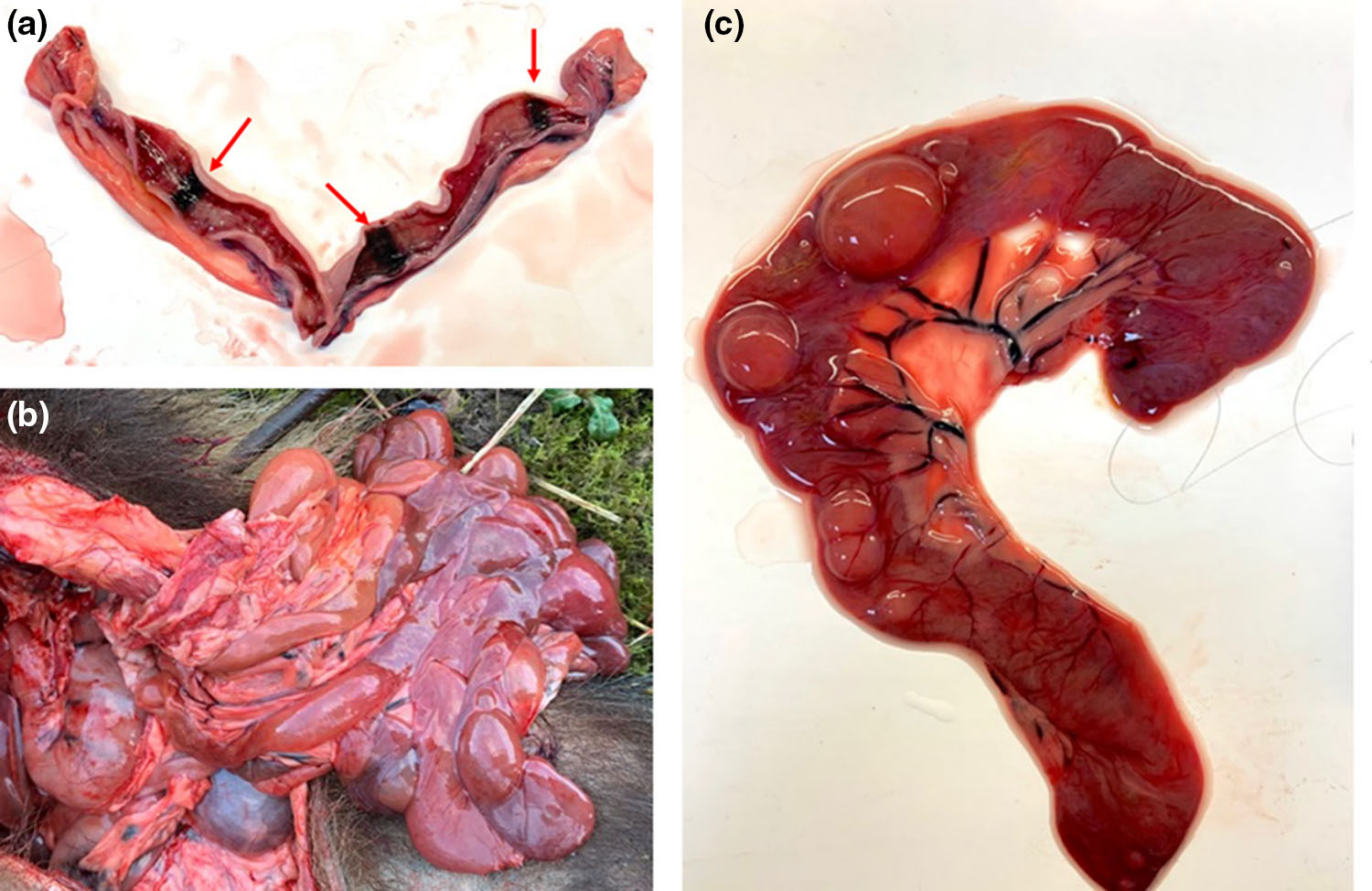
The fresh scars (red arrows) present in uterus of badger 1469 suggests that she had recently given birth to 3 cubs (**a**). The intestines of the badger were severely necrotic, enlarged and filled with gas (**b**). A closer look of the ileum (**c**) (Color figure online).

**Figure 4:**
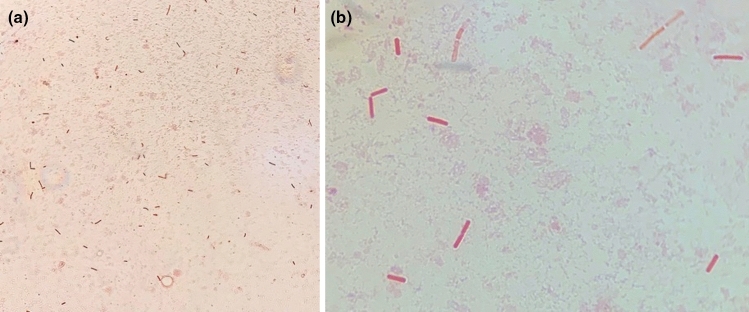
Gram stain revealed large amounts of rod-like gram-positive bacteria in the ileum mucous tentatively identified as *C. perfringens* under microscopic magnification of 40× (**a**) and 100× (**b**).

**Figure 5:**
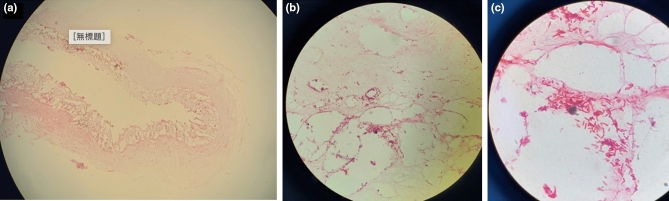
The histology pictures of the formalin-fixed ileum tissue at 4× (**a**) and gram-positive bacilli attached on lysed cells at 400× (**b**) and 1000× (**a**).

**Figure 6:**
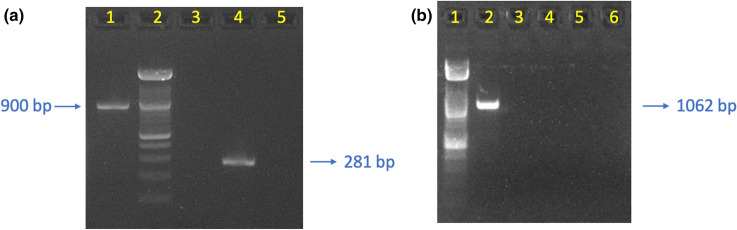
Gel images of the PCR amplification results of DNA extracted from 1469 s ileum tissue sample targeting various pathogen genes. **a** Lane1: multiplex PCR of *C. perfringens* toxins, only amplification of *cpa* gene is detected; lane 2: Ladders of DNA with 100 bp as interval; lane 3: amplification of canine circovirus *rep* gene; lane 4: amplification of MusGHV-1 DNA polymerase gene; lane 5: negative control with nuclease-free water as template. **b** Lane1: DNA ladder, lane 2: Multiplex PCR of *C. difficile* 16srDNA gene and toxin gene, only the *C. difficile* specific 16 s rDNA gene is detected; lane 3: amplification of *C. perfringens NetB* toxin gene; lane 4: amplification of C. perfringens *NetE* toxin gene; lane 5: amplification of *C. perfringens NetF* toxin gene; lane 6: amplification of *C. perfringens NetG* toxin gene.

From the postmortem results (Figs. 2, 3 and Supplementary file 1), we found that *C. perfringens* can proliferate in the badgers’ ileum (Fig. 4). However, given the duration over which the cadaver had been decomposing, it was impossible to determine whether necro-hemorrhagic enteritis was ante- or postmortem, and thus whether *C. perfringens* infection contributed to the cause of death of this individual, or whether this was solely autolysis leading to putrefaction (Fig. 5). Diagnosis of the pathophysiology arising from *C. perfringens* is very challenging because this pathogen is frequently present in the environment and often in the gut microbiome of healthy animals. Consequently, the differential elimination of competing pathogens from the diagnosis, combined with the application of multiple stands of symptomatic and clinical evidence, is required. Notably, however, at the final examination of this same individual in November 2019 (i.e., about 15 weeks before its death), as part of our long-term trapping and monitoring program (see Macdonald et al. 2015), the rectal swab we collected tested negative for *C. perfringens.* This suggests that the infection levels were either too low to be detected via conventional PCR or that the animal subsequently contracted this pathogen, perhaps fatally. However, the MusGHV-1 PCR sequencing product amplified from postmortem gut samples confirmed this individual carried the common genotype of MusGHV-1, which suggests that *C. perfringens* disease development may not be associated with the specific reactivated MusGHV-1 strain, despite being one of the risk factors for *C. perfringens* proliferation (Fig. 6).

Only one previous report has detected of *C. perfringens* infection in a European badger. The bacterium was isolated from the lung and spleen of a dead individual in Italy (Di Sabatino et al. 2016). The cause of death was diagnosed as canine distemper virus infection, with no discussion of any pathogenic role of *C. perfringens* (Di Sabatino et al. 2016). Pathologic infections with *C. perfringens* have been reported for other members of the Family Mustelidae, such as captive mink (*Mustela (*now *Neogale) vison*) (Macarie et al. 1980) and among captive breeding colonies of the highly endangered black-footed ferrets (*Mustela nigripes*) (Schulman et al. 1993). Wild Sea otters (*Enhydra lutris nereis*) are also susceptible to *C. perfringens,* where prevalence rates are 7.3 times higher among necropsied sea otters than live-sampled individuals (Miller et al. 2010). In captivity, several Carnivora species have been reported suffering from disease or mortality associated with *C. perfringens* type A. These include a group of cheetahs (*Acinonyx jubatus*) infected with *C. perfringens* enterotoxin (cpe), which recovered after treatment (Citino 1995); 1 African lion (*Panthera leo*) and 1 Amur tiger (*Panthera tigris altaica*) with fatal consequences in a zoo (Zhang et al. 2012); and 2 Amur leopards (*Panthera pardus orientalis*) although no *C. perfringens* genotyping was done in this case to confirm the diagnosis (Neiffer 2001).

Ours is the first report of any link between MusGHV-1 and *C. perfringens* occurrence, although this co-stressor of immunity is similar to other examples of coinfections with *C. perfringens* and other pathogens that can damage intestinal mucosa. Examples include CPV in dogs (Silva et al. 2017), coccidia in chickens (*Gallus gallus domesticus*) (Collier et al. 2008), nematodes in Hamadryas baboons (*Papio hamadryas*) (Nikolaou et al. 2009) and turkeys (*Meleagris gallopavo*) (Norton et al. 1992). Compromised host immunity generally is a risk factor, as infection with *C*. *perfringens* diminishes mature neutrophils in bone marrow, leading to lowered replenishment of mature neutrophils in the peripheral circulation, resulting in an innate immune deficiency of the host (Takehara et al. 2016; Van Lieshout et al. 2020). Indeed, any stressful conditions causing an adrenocortical response (Herman et al. 2016), such as high-density stocking in poultry (Tsiouris et al. 2015) and disruption of gut microbiome composition (Zaytsoff et al. 2020), pose potential risks. Other causes of *C. perfringens* enteritis include food poisoning (Grass et al., 2013) or the ingestion of feces infected with abundant *C. perfringens* cells, for example, from scavenging carcasses. In addition, a change to a high protein (Zentek et al. 2003) or high carbohydrate (Allison et al. 1975; Butler et al. 2008) diet can also predispose the gut to *C. perfringens* overgrowth.

Several studies report that gammaherpesvirus reactivation is associated with coinfection with other pathogens; for instance, EHV-2 was confirmed experimentally as a predisposing factor for *Rhodococcus equi* pneumonia in foals (Nordengrahn et al. 1996). Furthermore, more than 80% of infertile cows that tested positive for Bovine herpesvirus 4 also tested positive in pathogenic bacterial and/or fungal culture results, including for *C. perfringens* as one of the primary species (Chastant-Maillard 2015). In humans, a series of studies examining KSHV revealed that the pathogen-associated molecular patterns (PAMPs) produced by *Staphylococcus aureus* can promote virus entry, latency establishment and reactivation in the oral cavity of HIV-positive patients (Dai et al. 2014, 2020b). In addition, several recent studies have applied high throughput sequencing to search for microbiome signals to indicate EBV and KSHV reactivation (Gruffaz et al. 2020; Urbaniak et al. 2020). Experimentally, *Murid herpesvirus 4* primary infection has been established to sensitize mice to abortion induced by bacterial PAMPs, even at low doses (Cardenas et al. 2011). The above examples thus demonstrate that herpesvirus reactivation/viral shedding interacts closely with host microbiomes. Our results here imply a similar relationship between MusGHV-1 and *C. perfringens* in badgers, and stress-related MusGHV-1 reactivation may enhance shedding of this zoonotic bacterium into the environment. Generally, further investigation into disease causality and development is warranted to better understand the underlying mechanisms and consequences of infection. This will be informative for zoonotic disease management using the One Health approach in the interface between humans, domestic animals and wildlife.

## Supplementary Information

Below is the link to the electronic supplementary material.

Supplementary file1 (DOCX 24 KB)

Supplementary file2 (CSV 3 KB)

## Data Availability

All data generated or analyzed during this study are included in this published article as supplementary file 2.
