# Supplementary material for: Stress-Related Herpesvirus Reactivation in Badgers Can Result in Clostridium Proliferation
Source: Ecohealth. 2021 Dec 6;18(4):440–50. doi: 10.1007/s10393-021-01568-2 (PMC8742816; doi:10.1007/s10393-021-01568-2)
Supplement: Supplementary file 1 — Supplementary file1 (DOCX 24 KB) [file 10393_2021_1568_MOESM1_ESM.docx]

Supplementary Material: *Post-mortem report*

On 14^th^ March 2020, a badger was reported dead on a pasture adjacent to the River Thames, about 320 meters from the edge of Wytham Woods (Oxfordshire), c. 770 meters away from its sett of residency (Figure 2A). The carcass was very fresh and collected on 16^th^ March (average maximum outdoor temperature between 14^th^ - 16^th^ March was 11.7˚C and the minimum temperature was 2˚C, with no precipitation). This badger was identifiable from the tattoo on its inner thigh as belonging to the long-term study population monitored continuously at Wytham Woods since 1987 (Macdonald et al. 2015). This tattoo, ‘1469’ (Figure 2B), enabled the female to be matched to its previous trapping record: Since her birth in 2013, this female had been captured 7 times in total during her life, each time at the same social group. She showed signs of lactation in 2016 and 2018, but with no offspring assigned by genetic pedigree. A relatively large-bodied female, this badger had exhibited consistently thin body-condition throughout its capture history, although when last caught on 20^th^ Nov 2019 it was very fat, with a maximum body-condition score, weighing 9.9kg, with a minor healed wound on its rump. It was administered an intramuscular dose of BCG vaccination (Sofia) against bovine tuberculosis (bTB) infection.

A simple dissection and postmortem examination were conducted in the field to examine lungs, liver, kidneys, heart, stomach, intestine and reproductive system and ascertain possible cause of death. Uterine scars were observed, indicative of the animal having given birth to 3 cubs in the preceding spring (Figure 3A). The animal’s body condition was very poor with no subcutaneous fat. The stomach was completely empty, and no food content or nematodes were found throughout the rest of the gastrointestinal tract. The intestine, especially the ileum, was enlarged and loss of structural integrity, filled with gas, necrotizing and heamorrhagic on the surface (Figure 3B & 3C). Fresh samples of liver, lung, kidney and ileum were collected and stored at -20˚C for later laboratory examination. Part of the ileum was also preserved in 10% formalin for histological examination.

Microscopic examination of a gram stain slide of ileum mucosa revealed a predominance of gram-positive bacilli (Figure 4). Clinical presentation and bacterial morphology caused us to suspect that this heamorrhagic necrotic enteritis was caused by *C. perfringens*. We therefore performed a multiplex PCR to identify the genotype(s) (primer list in Table 1) of the pathogen involved, which confirmed presence of *C. perfringens* type A, where only the α toxin gene *cpa* was successfully amplified by PCR but no other toxin genes. We also performed PCRs to detect and differentiate other potential enteric pathogens including *Aleutian mink disease virus* (AMDV) (Jensen et al. 2011), *canine circovirus* (primers designed using canine circovirus replication associated protein gene), *mustelid gammaherpesvirus 1* (MusGHV-1) (King et al. 2004), *Clostridium difficile* (multiplex PCR to determine pathogenicity via detection of toxin gene A and B) (Persson et al. 2008), *Brucella spp.* (Mukherjee et al. 2007), *Salmonella spp.* (Sin et al. 2014), *Campylobacter spp.* (Yamazaki-Matsune et al. 2007) and *Mycobacterium spp.* (multiplex PCR to detect *M. tuberculosis* complex species, or other *Mycobacterium* species) (Mokaddas and Ahmad 2007). Only MusGHV-1 and *C. difficile* returned positive results, but *C. difficile* was determined to be non-pathogenic due to the absence of toxin genes A (tcdA) and toxin B (tcdB) and the binary toxin (cdtA/cdtB) genes (Figure 5). The PCR product of MusGHV-1 was then sent for genotyping using Sanger sequencing and classified as the common genotype.

The microscopic examination of histology shows cell autolysis of the villus and epithelium indicating severe postmortem changes (Figure 6, H&E stain), and large amount of gram-positive bacilli present in the edge of lysed cells (Figure 6, gram stain).

References:

Jensen TH, Christensen LS, Chriél M, Uttenthal Å, Hammer AS (2011) Implementation and validation of a sensitive PCR detection method in the eradication campaign against Aleutian mink disease virus. J Virol Methods 171:81–85 . https://doi.org/10.1016/j.jviromet.2010.10.004

King DP, Mutukwa N, Lesellier S, Cheeseman C, Chambers MA, Banks M (2004) Detection of Mustelid Herpesvirus-1 Infected European Badgers (Meles meles) in the British Isles. J Wildl Dis 40:99–102 . https://doi.org/10.7589/0090-3558-40.1.99

Mokaddas E, Ahmad S (2007) Development and evaluation of a multiplex PCR for rapid detection and differentiation of Mycobacterium tuberculosis complex members from non-tuberculous mycobacteria. Jpn J Infect Dis 60:140–144

Mukherjee F, Jain E, Patel V, Nair M (2007) Multiple genus-specific markers in PCR assays improve the specificity and sensitivity of diagnosis of brucellosis in field animals. J Med Microbiol 56:1309–1316 . https://doi.org/10.1099/jmm.0.47160-0

Persson S, Torpdahl M, Olsen KEP (2008) New multiplex PCR method for the detection of Clostridium difficile toxin. A (tcdA) and toxin. B (tcdB) and the binary toxin (cdtA/cdtB) genes applied to a Danish strain collection. Clin Microbiol Infect 14:1057–1064 . https://doi.org/10.1111/j.1469-0691.2008.02092.x

Sin YW, Annavi G, Dugdale HL, Newman C, Burke T, MacDonald DW (2014) Pathogen burden, co-infection and major histocompatibility complex variability in the European badger (Meles meles). Mol Ecol 23:5072–5088 . https://doi.org/10.1111/mec.12917

Yamazaki-Matsune W, Taguchi M, Seto K, Kawahara R, Kawatsu K, Kumeda Y, Kitazato M, Nukina M, Misawa N, Tsukamoto T (2007) Development of a multiplex PCR assay for identification of Campylobacter coli, Campylobacter fetus, Campylobacter hyointestinalis subsp. hyointestinalis, Campylobacter jejuni, Campylobacter lari and Campylobacter upsaliensis. J Med Microbiol 56:1467–1473 . https://doi.org/10.1099/jmm.0.47363-0
